# The Biological Activities and Therapeutic Potentials of Baicalein Extracted from *Oroxylum indicum*: A Systematic Review

**DOI:** 10.3390/molecules25235677

**Published:** 2020-12-02

**Authors:** Nik Nur Hakimah Nik Salleh, Farah Amna Othman, Nur Alisa Kamarudin, Suat Cheng Tan

**Affiliations:** School of Health Sciences, Health Campus, Universiti Sains Malaysia, Kubang Kerian 16150, Kelantan, Malaysia; nikhakimahabdullah@yahoo.com (N.N.H.N.S.); farahamna88@gmail.com (F.A.O.); isa.addeen@gmail.com (N.A.K.)

**Keywords:** *Oroxylum indicum*, baicalein, medicinal plant, biological activities, therapeutic potential

## Abstract

In Southeast Asia, traditional medicine has a longestablished history and plays an important role in the health care system. Various traditional medicinal plants have been used to treat diseases since ancient times and much of this traditional knowledge remains preserved today. *Oroxylum indicum* (beko plant) is one of the medicinal herb plants that is widely distributed throughout Asia. It is a versatile plant and almost every part of the plant is reported to possess a wide range of pharmacological activities. Many of the important bioactivities of this medicinal plant is related to the most abundant bioactive constituent found in this plant—the baicalein. Nonetheless, there is still no systematic review to report and vindicate the biological activities and therapeutic potential of baicalein extracted from *O. indicum* to treat human diseases. In this review, we aimed to systematically present in vivo and in vitro studies searched from PubMed, ScienceDirect, Scopus and Google Scholar database up to 31 March 2020 based on keywords “*Oroxylum indicum*” and “baicalein”. After an initial screening of titles and abstracts, followed by a full-text analysis and validation, 20 articles that fulfilled all the inclusion and exclusion criteria were included in this systematic review. The searched data comprehensively reported the biological activities and therapeutic potential of baicalein originating from the *O. indicum* plant for anti-cancer, antibacterial, anti-hyperglycemia, neurogenesis, cardioprotective, anti-adipogenesis, anti-inflammatory and wound healing effects. Nonetheless, we noticed that there was a scarcity of evidence on the efficacy of this natural active compound in human clinical studies. In conclusion, this systematic review article provides new insight into *O. indicum* and its active constituent baicalein as a prospective complementary therapy from the perspective of modern and scientific aspect. We indicate the potential of this natural product to be developed into more conscientious and judicious evidencebased medicine in the future. However, we also recommend more clinical research to confirm the efficacy and safety of baicalein as therapeutic medicine for patients.

## 1. Introduction

**Figure 1:**
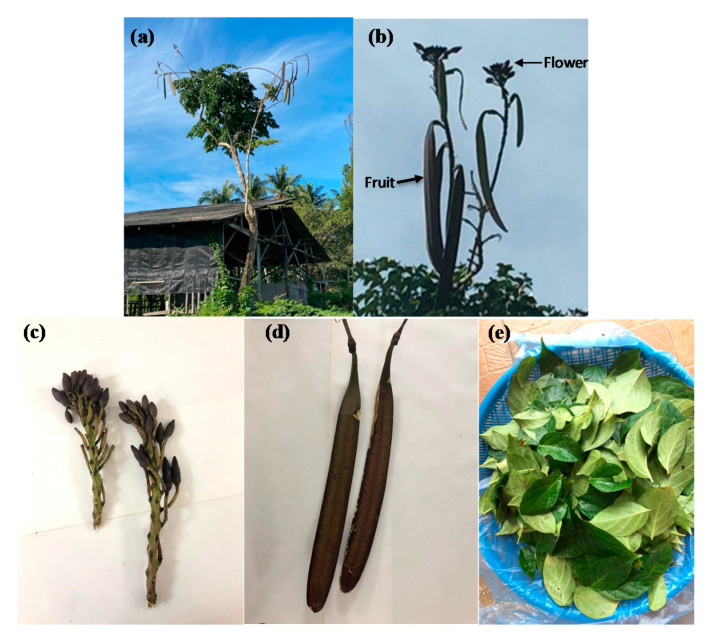
Representative images of *O. indicum* plant. (**a**) *O. indicum* is a medium-sized tree. (**b**) The flowers bloom on top of the tree and the fruits hang down from the bare branches like dangling swords. (**c**) The flowers are numerous in numbers, reddish purple outside and pale, pinkish-yellow. (**d**) The fruits are woody, winged, large, flat and sword shaped. (**e**) The leaves are pinnately compound, ovate-elliptic with acuminate tips.

*Oroxylum indicum* (L.) Benth. ex Kurz. is a medicinal herb plant belonging to family Bignoniaceae that is widely distributed throughout Asian regions, including India, Thailand, Vietnam, Malaysia, Indonesia, Philippines, China, Taiwan, and Japan [1,2]. It is a small to medium-sized, lanky, and fast growing deciduous tree that can grow up to 8–15 m tall in general (Figure 1a). Bark of the plant is light greyish brown in colour and soft, spongy in texture with corky lenticels. The flowers are numerous in numbers, reddish purple outside and pale, pinkish-yellow within which emit a strong, fetid odour to attract pollinators and they form enormous seed pods (fruits) that hang down from bare branches (Figure 1b,c). The fruits are woody, winged, large, flat and sword shaped (Figure 1d). The seeds present within fruit are round, flat and thin with broad silvery papery wings except at the base. The leaves are pinnately compound which normally grows up to 3–10 cm long with 2–4 leaflet pairs and they are evergreen (Figure 1e).

*O. indicum* is regarded as a dual-purpose plant (can be consumed as food or taken as traditional medicine remedy) and has been used widely throughout Southeast Asia for decades. In Malaysia, *O. indicum* is commonly known as “beko”. It is a locally popular vegetable which often sells in markets for its leaves and fruits that are consumed as raw salad (ulam) by the local populations. *O. indicum* has been consumed by the local population as daily meal even long before it has been reported to exert anti-aging properties and improve one’s health [3]. *O. indicum* is a versatile plant and possesses wide range of biological and pharmacological activities. Every part of the plant, including its bark, leaves, fruits, and seeds, is reported to have a broad array of biological activities and has already been applied in complementary medicine to treat human diseases [1,2].

### Baicalein—The Major Chemical Compound Found in O. indicum

**Figure 2:**
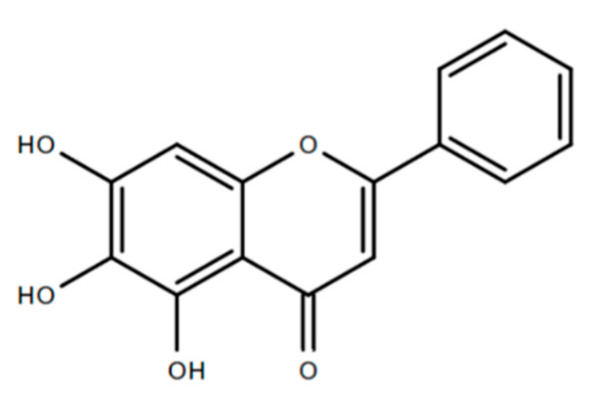
Chemical structure of baicalein compound.

The biological activities reported in different parts of the *O. indicum* plant are mediated by a broad range of secondary metabolites, such as flavonoids, alkaloids, tannin, glycosides, saponin, phenols and quinones found in the *O. indicum* plant [4,5,6,7,8]. Among them, flavonoids are the major storage components of *O. indicum* which could be found in almost all parts of the plant using various types of extraction solvents including methanol, ethanol, chloroform, water, and ethyl acetate [4,8,9,10,11,12,13]. The flavonoids constituents present in *O. indicum* includes baicalein, baicalein-7-*O*-glucosice, baicalein-7-*O*-diglucoside, chrysin, and oroxylin-A [14]. Among all of these flavonoids, baicalein is the most abundantly found and dominant active compound of the *O. indicum* plant in general [9,14,15]. Numerous studies conducted on phytochemical investigations of *O. indicum* clearly demonstrated the abundance of baicalein isolated from various parts of this plant including the stem barks, root barks, leaves, fruits and also seeds [4,5,6,7,8]. Baicalein (C_15_H_10_O_5_), also chemically known as 5,6,7-trihydroxyflavone, is a member of flavonoids compound, under flavones sub-groups with a structure based on the backbone 2-phenylchromen-4-one (2-phenyl-1-benzopyran-4-one) (Figure 2).

Being the amplest flavonoid present in *O. indicum*, baicalein is strongly related to many of the plant’s biological activities as reported in numerous pharmacological reports. Nonetheless, to our knowledge, there is still no systematic review to report and vindicate the biological activities and therapeutic potential of baicalein extracted from *O. indicum* to treat human ailments. Therefore, in this study, we aimed to use the Preferred Reporting Items for Systematic Reviews and Meta-Analyses (PRISMA) guideline to comprehensively assess the biological properties and the efficacy of baicalein extracted from this plant for the treatment of various human diseases as described in Section 2.

## 2. Methodology

### 2.1. Search Strategy

In this review, a systematic literature search based on PRISMA guideline was performed to identify publications studied the effects of baicalein extracted from *O. indicum* for the treatment of various diseases conducted in vitro, in vivo or in both.

### 2.2. Research Article Selection and Evaluation

Search results were limited to fully documented articles in English following the inclusion and exclusion criteria as listed below:

Inclusion Criteria:

- In vitro studies related to baicalein extracted from *Oroxylum indicum*
- In vivo studies related to baicalein extracted from *Oroxylum indicum*
- Intervention clinical study with baicalein extracted from *Oroxylum indicum*
- Full-text articles

Exclusion criteria:

- Irrelevant titles and abstracts
- Duplicated studies
- Review articles/meta-analyses
- News/editorials/letters
- Case reports
- Baicalein extracted from other medicinal plant
- Synthetic/commercialized baicalein
- Non-English language

Two independent reviewers screened the articles based on the inclusion and exclusion criteria stated above. Next, the remaining papers were checked for duplications and those with exclusion criteria were also eliminated. For the first screening, the related articles were screened based on their titles and abstracts. Finally, the selected full-text articles were checked by another reviewer according to the inclusion criteria for final validation.

## 3. Results

**Figure 3:**
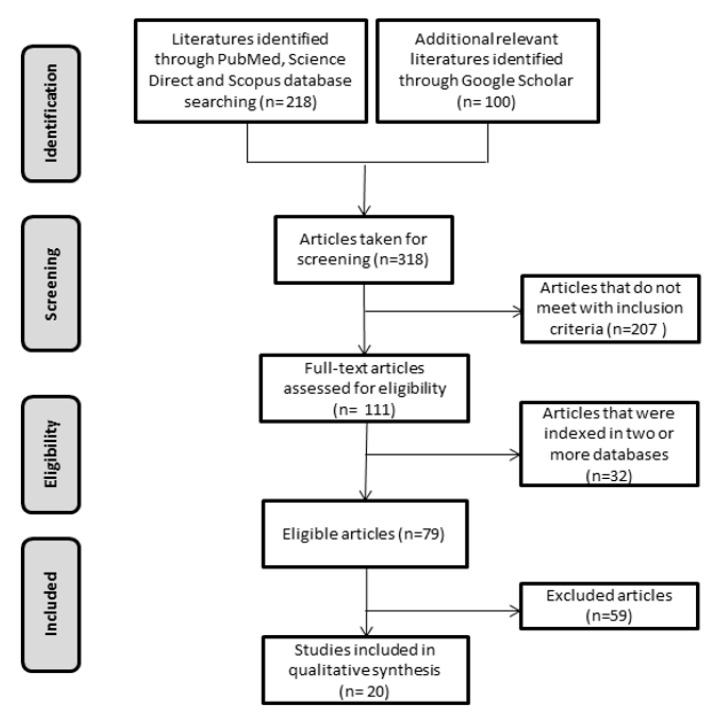
Flowchart illustrating the progressive study selection based on PRISMA Flow Diagram.

Study selection: The primary search identified 218 articles, including 31 from PubMed, 119 from Science Direct and 68 from Scopus. First 100 hits from the ‘Most Relevant’ option in Google Scholar were also added. Among these, 207 documents were published in languages other than English or not a research article and were therefore excluded from the review. In addition, 32 manuscripts were indexed in two or more databases and were considered only once, resulting in 79 eligible articles. After an initial screening of titles and abstracts, followed by a full text analysis and validation, 20 articles were included in this systematic review. The year of publication for each of these articles is listed in Table 1. A flowchart illustrating the progressive study selection and numbers at each stage based on PRISMA guideline is shown in Figure 3.

## 4. Discussion

### 4.1. Biological Activities of Baicalein Extracted from O. indicum

**Figure 4:**
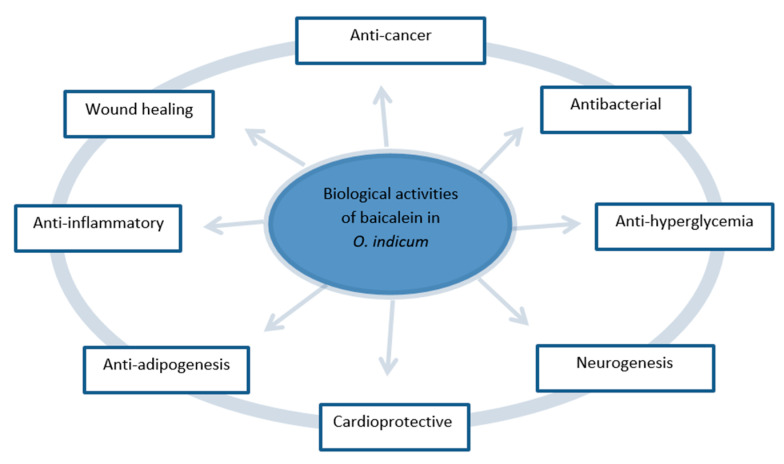
The major biological activities of baicalein extracted from *O. indicum* medicinal plant.

The selected articles were carefully read and analysed. Based on the findings reported in these articles, we identified eight major biological activities exerted by the baicalein extracted from *O. indicum* (Figure 4) and discussed in detail in the following section. The summary of all key findings is listed in Table 2.

#### 4.1.1. Anti-Cancer

Cancer is one of the leading causes of death worldwide. Nonetheless, the high cost of cancer treatment implies a major constraint for patients from low-income countries to have a good access to high quality healthcare. These populations often turn towards the use of relatively more affordable traditional complementary medicine. In 2018, Chassagne et al. [16] employed a bibliographic scoring approach to select traditional plants frequently used in the treatment of liver disorders in Southeast Asia and tested their anti-cancer activities on liver cancer (HepG2 cell line). There were 10 plants tested in the study, including *O. indicum*, *Andrographis paniculata* (Burm.f.) Nees, *Willughbeia edulis* Roxb, *Senna alata* (L.) Roxb., *Cananga latifolia* (Hook.f. & Thomson) Finet & Gagnep., *Salacia chinensis* L., *Orthosiphon aristatus* (Blume) Miq., *Boerhavia diffusa* L., *Gomphrena celosioides* Mart. and *Melastoma saigonense* (Kuntze) Merr. Interestingly, when compared to the other plants evaluated in the study, the ethanolic extract of *O. indicum* showed the highest anti-proliferative effect on the HepG2 model (IC_50_ = 64.1 μg/mL). It was speculated that its anti-proliferative effect could be attributed to the presence of flavonoids including baicalein which had been previously reported to exert anti-proliferative effects on liver cancer. This study is supported by several other studies on the anti-cancer properties of baicalein extracted from *O. indicum* against several other types of human cancer cells in vitro.

Firstly, according to Yang et al. [17], baicalein treatment at doses of 5 mg/mL for 72 h inhibits bladder cancer cell viability via a marked reduction of anti-apoptotic genes such as B-cell lymphoma 2 (BCL2), B-cell lymphoma extra large (Bcl-xL), X-linked inhibitor of apoptosis protein (XIAP) and survivin. Bcl-2, encoded by the BCL2 gene, is a regulator protein that induces apoptosis (pro-apoptotic protein). Bcl-2 and its pro-survival relative, Bcl-xL, are known to protect cells from apoptosis [18]. Moreover, XIAP and survivin are members of the inhibitor of apoptosis (IAP) protein family that inhibit caspases and block cell death. These proteins are highly expressed in most types of cancer cell and are associated with a poor clinical cancer treatment outcome [19]. The overexpression of these four proteins in cancer cells may block or delay the onset of apoptosis by selecting and maintaining the long-living cancer cells in the G0 phase of the cell cycle. Therefore, the ability of baicalein treatment to mediate the reduction of these anti-apoptotic gene expressions in bladder cancer cells as reported by Yang et al. [17] could be explored as a therapeutic potential for the development of cancer treatment strategy.

Baicalein has also been investigated for its inhibitory effects against glioblastoma multiforme (GBM) brain cancer by Kang et al. [11]. The research group extracted crude compounds from *O. indicum* leaves using binary extraction system composed of a combination of petroleum ether and methanol solvents to generate crude extract with 15% of baicalein content. In order to further enhance the baicalein content in the crude extract, it was subjected to an enrichment fractionation chromatography composed of Diaion HP20 resin column and methanol solvent which successfully increased the baicalein content by nearly two-fold (from 15% to 29%). The baicalein enriched extract obtained after the fractionation chromatography not only contained higher baicalein concentration, it also possessed higher antioxidant activity as well as enhanced efficiency to inhibit GBM cancerous cell growth (IC_50_ baicalein-enriched fraction = 36 μg/mL vs. IC_50_ crude extract = 92 μg/mL). Therefore, it can be logically hypothesized that the major phytochemical of *O. indicum* that confers the anti-GBM medicinal benefits is the baicalein. The fascinating effects of the baicalein active compound on GBM cell inhibitory could be due to its major capacities to inhibit nuclear factor-kappa B (NF-kB), a multifunctional transcriptional nuclear factor which involves extensively in cancer development and progression [20]. Attenuation of the NF-kB pathway has long been linked to inhibit various types of cancers such as ovarian cancer [21], gastric cancer [22], and breast cancer [23]. Moreover, baicalein also has been proved to induce apoptosis by activating caspase-9/-3 and to inhibit tumor invasion and metastasis by reducing the expression of matrix metalloproteinase-2/-9 (MMP-2/-9) [24]. In addition, baicalein possesses a unique feature to cross blood-brain barrier (BBB), enabling its distribution over a wide range of brain area after oral administration [25]. This feature has significantly supported its therapeutic potential for brain and central nervous system (CNS) diseases, including the brain cancer.

Furthermore, in another study done by Wahab et al., baicalein-rich fraction extracted from *O. indicum* leaves was found to attenuate the growth and survival of cervical cancer cells greater than crude extract with less baicalein content by down-regulating the expression of E6 and E7 oncoproteins that play a key role in human papillomavirus (HPV)-associated cervical carcinoma tumorigenesis [26]. HPV E6 oncoprotein targets p53 tumor suppressor protein and inhibits its roles to initiate DNA repair and apoptosis signalling pathway. On the other hand, HPV E7 protein targets another tumor suppressor protein known as retinoblastoma protein (pRb) and inhibits its roles in regulating cell cycle and stabilizing cell proliferation/cell death balance [27]. Taken together, the downregulation of E6 and E7 proteins and restoration of p53 and pRb proteins by baicalein could be a promising therapeutic approach to block excessive cell growth, a hallmark of cancerous cells. These findings were supported by Zazali et al. which also reported an anti-proliferative activity of methanolic leaf extract of *O. indicum* against cervical cancer (HeLa cell line) by inducing G1/S cell cycle arrest and apoptosis via p53-mediated pathway [28]. They found that *O. indicum* methanol extract which consisted of baicalein caused high accumulation of p53 protein in HeLa cells (up to 88.5%) after 72 h treatment at concentration of IC_50_ (3.87 μg/mL). It is well known that p53 functions as a transcription factor for genes involve in the regulation of normal cell cycle [29]. After 72 h of treatment, the DNA amount of HeLa cells in G0/G1 phase raised up to 99.31% while the DNA amount in both S phase and G2/M phase dramatically reduced to 0.7% and 0.08% only. This indicated that the growth of HeLa cells was impeded at the G1/S checkpoint, causing the interruption of cancer cells to enter the S phase to synthesis the DNA and thus, no excessive cancer cell growth occurred. Furthermore, p53 may also promote apoptosis of cancerous cells. This was proved by the increment of early and late apoptotic cells after 48 and 72 h of treatment with *O. indicum* extract in the study [28].

Last but not least, there was a study that reported that baicalein exhibited the most potent inhibitory effects on growth and metastasis of human colorectal cancer cells compared to other flavonoids isolated from the same *O. indicum* stem bark extracts, included the chrysin, oroxylin-A, oroxylin-A glycoside and baicalin [30]. The study also described the role of baicalein as an inhibitor of kexin-type proteases of superfamily proprotein convertase subtilisin/kexins 3 (PCSK3) which is also known as furin. Furin has been well documented to cleave and convert inactive growth factors into their matured forms that promote tumor growth. The role of furin in cancer progression renders it an attractive target for cancer treatment. There are many on-going efforts to identify furin inhibitors to inhibit cancer progression to a significant extent. There were reports in the literature which showed that PCSK inhibitors could significantly decrease the proliferation, tumorigenesis and metastasis of skin cancer cells [31], colorectal cancer cells [32] and breast cancer cells [33]. These findings justify and rationalize the potential to explore baicalein as a new lead natural furin inhibitor for future cancer therapy.

#### 4.1.2. Antibacterial

Emerging evidence has shown that that the extracts from *O. indicum* promoted moderate to intermediate antibacterial activities against clinically isolated bacteria such as *Streptococcus suis* and *Staphylococcus intermedius* in a dose-dependent manner. In a study done by Sithisarn et al. [7], *O. indicum* fruits were collected from different provinces in Thailand and the bioactive compounds were extracted using 95% ethanol and distilled water, respectively. Both extracts were determined for their in vitro antibacterial effects on two clinically isolated bacteria, *S. suis* and *S. intermedius*, using disc diffusion assay. They found that the extracts prepared by maceration with ethanol promoted higher antibacterial activities than those prepared with water. Phytochemical characterization analysis of both extracts detected a chromatographic band that could correspond to flavonoid baicalein. A further in vitro DPPH scavenging assay also confirmed that both extracts exhibited high antioxidant effects due to the high total flavonoid contents in both extracts.

In their subsequent study, they described further detail regarding the effects of different extraction methods (ethanol extract vs. water extract) on the antibacterial activities [12]. As reported, the half maximal inhibitory concentrations (IC_50_) of *O. indicum* fruits ethanol extract against four types of clinical isolated bacteria namely: *S. intermedius, S. suis, P. aeruginosa* and *E. coli* were 1.30, 7.81, 39.20, and 66.85 mg/mL respectively, while the IC_50_ of *O. indicum* fruits water extract against these four types of bacteria were 7.81, >250, >125, and >250 mg/mL respectively. This indicated that ethanol extract showed higher potential as an antibacterial agent than the water extract. This was because the flavonoid content (including baicalein) was found to be significantly higher in the ethanol extract compared to water extract (based on the optimized and validated HPLC data). In brief, their findings highlighted that the antibacterial potential of *O. indicum* extract was highly correlated to the biological activities of flavonoids such as baicalein. The higher the baicalein content, the higher the antibacterial potential exhibited by the *O. indicum* extracts.

#### 4.1.3. Anti-Hyperglycemia

Diabetes mellitus (DM) is characterized by high levels of glucose in blood (hyperglycemia). The effect of baicalein on glucose uptake and insulin sensitivity to prevent hyperglycemia was investigated by Singh and Kakkar [34] who reported that 50% aqueous ethanolic *O. indicum* stem bark which contained baicalein showed anti-hyperglycemia effects in vitro (3T3-L1 adipocytes cell line) and in vivo (diabetic rat models) via the inhibition of α-glucosidase activity. The main function of α-glucosidase enzyme is to catalyze hydrolysis of dietary carbohydrates and starches to produce glucose for intestinal absorption [35]. Therefore, inhibiting the function of these enzymes may delay the glucose production following dietary digestion, which in turn reduces hyperglycemia. In the study, it was found that *O. indicum* extract exhibited α-glucosidase inhibition effect comparable to clinically used standard α-glucosidase inhibitor known as acarbose [34]. This finding was further supported by another study which reported that administration of 4 mg/kg acarbose (low dose) combined with 200 mg/kg *O. indicum* seed extract showed a blood glucose lowering effect in diabetic mice similar to animal group that received 20 mg/kg of acarbose (high dose) single treatment only [36]. These data indicated that *O. indicum* extract could enhance the efficacy of acarbose by up to five-fold (as it could reduce acarbose dosage from high dose 20 mg/kg to low dose 4 mg/kg) when injected simultaneously (combined *O. indicum* + acarbose) into diabetic mice. Moreover, the synergistic effect of combined *O. indicum* + acarbose treatment also was observed in prediabetic mice model as reported by Sun et al. [36]. Prediabetes is defined as blood glucose levels above normal level but yet still below the diabetes threshold. It is an initial period of type 2 DM and up to 70% of individuals with prediabetes will eventually develop diabetes if left untreated [37]. The pathophysiological defect seen in prediabetes can be managed with lifestyle and diet modifications, thus it is essential to have a clearer understanding of the benefit of herbs diet such as *O. indicum* in preventing the progression from prediabetes to diabetes. In the study done by Sun et al., the effects of acarbose and *O. indicum* seed extract combination on glucose tolerance and diabetic complications were investigated in rat models at initial period of type 2 DM (the prediabetic model). The combination treatment showed higher synergistic hypoglycaemic activity than acarbose single treatment by reducing 80% of the acarbose dose and 75% of the diabetes risk [36].

The synergistic effect of the combined *O. indicum* and acarbose treatment is beneficial as this could reduce the toxicity side effects caused by large doses of single-use pharmaceutical acarbose drug in diabetes management. Although acarbose was the first efficient drug approved by the Food and Drug Administration (FDA) for diabetic and pre-diabetic treatment, it was recently reported associated with several adverse effects. Hepatic side effects are the major side effects of acarbose [38]. Moreover, it also can cause allergic or chronic gastrointestinal disorders due to diarrhea and flatulence that may deteriorate to hernia, intestinal obstruction or enterelcosis [39]. Sun et al. reported that the combined *O. indicum* + acarbose treatment which has low toxicity could hinder the side effects of increased alanine aminotransferase (ALT) as seen in the acarbose single treatment group [36]. ALT is an enzyme found mostly in the liver and kidney. Normally, ALT levels are low in blood, but when the liver is damaged, ALT is released into the blood and the level increases. Therefore, the detection of ALT level in blood is a reliable test for early detection of liver disease. Microscopically, the study also revealed that the combined treatment group has little liver cellular swelling or hepatic steatosis as compared to acarbose single treatment group, indicating that the combined drugs have the potential to restore and repair the liver structure and function significantly [36].

Furthermore, besides mimicking the function of acarbose, baicalein also could treat hyperglycemia via the inhibition of bovine serum albumin (BSA) glycation [34]. BSA glycation is a complex cascade of reactions in which the protein structure is altered by reducing sugar such as glucose. Glycation rate is commonly increased in diabetes patients due to excess glucose molecules presence in serum. Glycation may result in non-functional proteins and further trigger various signalling pathways leading to insulin resistance and diabetic complications. Moreover, further glycation may result in the formation of advanced glycation end products (AGEs) which could elevate the risk for beta cell injury and insulin resistance [40] and this situation in turn further increases excess glucose level and accelerates the AGE formation, thus forming a vicious cycle that worsen the hyperglycemic condition [41]. Therefore, the significant effect of *O. indicum* extract to inhibit BSA glycation as reported by Singh and Kakkar [34] can be a boon in anti-hyperglycemia management by protecting beta cells and improving insulin sensitivity.

Last but not least, in diabetes patients, the total antioxidant defenses are reduced due to the generation of oxygen free radical from glycosylation, auto-oxidation of glycation products and impairment of the endogenous antioxidant defense system [42]. Therefore, another possible mechanism of anti-hyperglycemia exerted by baicalein could be via its high antioxidant capacity which could eliminate the deleterious effects of oxidative stress accumulated in diabetes patients. This was reported by Sun et al. whose research indicated that therapeutic approaches using *O. indicum* extract rich in baicalein can attenuate the damage from oxidation reactions and were effective for the prevention of complications from prediabetes and diabetes conditions [36].

#### 4.1.4. Neurogenesis

Neurogenesis is the process by which neurons or nerve cells are generated in the brain via differentiation of neural stem cells (NSCs). Differentiation of NSCs into neurons is induced by basic helix-loop-helix (bHLH) activators such as neurogenin 2 (Ngn2) [43]. In a screening study using a cell-based luciferase assay system done by Fuentes et al., the *O. indicum* bark methanol crude extract successfully activated the Ngn2 promoter activity by two-fold, indicating its potential to induce neurogenesis [44]. Subsequently, the researcher isolated seven major active constituents from the crude extract of *O. indicum* using an activity-guided approach. The constituents extracted included oroxyin A, chrysin, hispidulin, baicalein, apigenin, baicalin and isoverbascoside. Among these compounds, baicalein showed a two-fold increase in Ngn2 promoter activity at 10 uM which correlated with the biological activities of the crude extract. In brief, their findings clearly highlighted that the neurogenesis potential of *O. indicum* extract was highly correlated to the baicalein.

#### 4.1.5. Cardioprotective

In vivo study by Menon et al. focuses on cardioprotective activity of 70% methanolic extract of *O. indicum* root bark against doxorubicin induced cardiomyopathy rats [45]. Doxorubicin is an anthracycline antibiotic used in chemotherapy for hematological malignancies and some types of tissue sarcomas and carcinomas, but it is reported to cause side effects such as heart damage through oxidative stress, mitochondrial dysfunction, increase the susceptibility of cardiac tissue to lipid peroxidation and reduce levels of antioxidant defences associated with the heart tissue. In that study, doxorubicin treated animals were found to exhibit elevated levels of cardiac injury markers such as lactate dehydrogenase (LDH), creatine phosphokinase (CPK), serum glutamate oxaloacetate transaminase (SGOT) and serum glutamate pyruvate transaminase (SGPT) and exhibited arrhythmia when examined using electrocardiogram (ECG). Interestingly, after the doxorubicin-treated animals were given the treatment using extract from root bark of *O. indicum*, all the cardiac injury markers were successfully reduced and the animals resumed back to normal ECG pattern. Furthermore, the cardiac tissue histological examination also showed amelioration of myofibrillar disorganization, reduced focal loss of tissue and fragmentation, lesser necrotic bodies, scattered cytoplasmic vacuolation and decreased lymphocyte infiltration in *O. indicum* treated group. The protective effects of *O. indicum* root bark extract on doxorubicin induced cardiomyopathy rats may be credited to its ability in enhancing the tissue antioxidant status, indicated by the significant (*p* < 0.01) elevation of cardiac tissue antioxidant profile in *O. indicum* treated group in a dose-dependent manner. These findings presented the capability of this plant in shielding the damaging and devastating effects of cumulative administration of doxorubicin in murine models.

#### 4.1.6. Anti-Adipogenesis

Adipocyte formation, also known as adipogenesis, plays a central role in the development of obesity. Tanaporn et al. [46] reported that ethanol extract of *O. indicum* fruit pods which contained baicalein was able to inhibit lipid and carbohydrate accumulation in adipocytes. Treatment of 200 μg/mL *O. indicum* ethanolic extract significantly decreased the intracellular lipid accumulation by approximately 52%, compared to the non-treated adipocyte (control). Staining with Oil Red O and hematoxylin revealed that untreated adipocytes displayed an increase in size and number of prominent lipid droplets while *O. indicum* extract-treated adipocytes exhibited smaller size and reduced number of lipid droplets. Furthermore, the study also found that *O. indicum* extract showed potential to inhibit pancreatic lipase activity. Pancreatic lipase is an enzyme to mediate the digestion of dietary fats (triglycerides), the uptake of fats into various tissues, and the mobilization of fats inside cells. Dietary triglycerides must be cleaved into free fatty acids and monoacylglycerols before they are absorbed by cells. Thus, when lipase activity is inhibited, it will lead to a decrease in the uptake of lipids into the human body (anti-adipogenesis effect) [47]. More importantly, the researchers also confirmed the non-toxicity effect of this extract on pre- and mature adipocytes at low doses (50–200 ug/mL), assuring the safety of taking this traditional supplement as a diet plan for the management of overweight or obese.

A subsequent research done by Tanaporn et al. further investigated the potential of *O. indicum* ethanolic extract on adiponectin secretion and explored the mechanism underlying the anti-adipogenesis effects exerted by the *O. indicum* ethanolic extract [46]. Adiponectin is a protein hormone involves in regulating glucose levels as well as fatty acid breakdown. This hormone also plays significant roles in the proliferation and differentiation of adipocytes by increasing the lipid accumulation and glucose transportation in adipocytes. The study found that 200 μg/mL *O. indicum* methanolic extract treatment was able to significantly lower the level of adiponectin by 2-fold as compared to the non-treated group. Decreased circulating adiponectin levels have been found to suppress the differentiation of 3T3-L1 pre-adipocytes to adipocytes. In addition, the researchers also proved that the *O. indicum* extract exerted anti-adipogenesis effects via the downregulation of peroxisome proliferator-activated receptor-gamma 2 (PPARγ2), an adipocyte-specific nuclear hormone receptor which plays a crucial role in the differentiation of pre-adipocyte to mature adipocyte; sterol regulatory element-binding transcription factor 1c (SREBP-1c), a transcription factor which is required in fatty acid metabolism and de novo lipogenesis; fatty acid synthase (FAS), an enzyme which is the crucial for intracellular triglyceride synthesis; glucose transporter type 4 (GLUT-4), a glucose carrier protein which facilitates glucose transportation into the cells and last but not least, the leptin, a hormone which regulates appetite, food intake and body weight [48]. The inhibition of all these five key factors has been found to significantly prevent the adipogenic processes in 3T3-L1 cells. These results are the indicators that baicalein in *O. indicum* possess the anti-obesity potential and may be developed as a novel natural product-based product for obesity prevention.

In another independent but related study, Mangal et al. expanded their research to six important Indian Ayurvedic medicinal plant species to identify the most potential active constituents as remedy against complications affecting glucose and lipid homeostasis [48]. The plants involved in the study were: *Limonia acidissima* Groff (Kapittha), *Cassia siamea* Lam. (Kassod), *Swertia chirayita* (Roxb.) Buch.-Ham. (Kiratatikta), *Oroxylum indicum* (L.) Kurz (Shyonaka), *Carissa carandas* Linn. (Karamarda) and *Capparis decidua* (Forssk.) Edgew. (Karir). Out of these six plant species, *O. indicum* which contained baicalein showed the highest pancreatic lipase inhibitory activity and lowest lipid accumulation in 3T3-L1 adipocyte culture, indicating the great potential of this plant to be developed as herbs against obesity.

#### 4.1.7. Anti-Inflammatory

Traditional medicine is widely used among the old folk generation since ancient times for anti-inflammation effects, especially to reduce redness, swelling, and pain on skin or open wounds. Nonetheless, this practice is seldom assessed scientifically in spite of their long history of uses for diminishing inflammatory conditions. Therefore, Siriwatanametanon et al. conducted a research to assess nine traditional plants with uses linked to anti-inflammatory as recorded in Thailand formal textbook [49]. The plants involved in the study were *Basella alba, Basella rubra* (Basellaceae), *Cayratia trifolia* (Vitaceae), *Gynura pseudochina* var. hispida, *Gynura pseudochina* (Asteraceae), *Muehlenbeckia platyclada* (Polygonaceae), *O. indicum* (Bignoniaceae), *Pouzolzia indica* (Urticaceae), and *Rhinacanthus nasutus* (Acanthaceae). Among all the nine species, *only Gynura pseudochina* var. hispida and *O. indicum* showed the most promising NF-κB inhibitory effects with the lowest IC_50_ values (41.96 and 47.45 μg/mL, respectively). Moreover, *O. indicum* also showed a high level of antioxidant activity by inhibiting lipid-peroxidation (IC_50_ 0.08 μg/mL). Similarly, another research group in Vietnam also performed a screening study of 17 medicinal plants used in Vietnamese traditional medicine for the treatment of inflammatory disorders [15]. The 17 plants were *Abutilon indicum* (L.) Sweet, *Achyranthes aspera* L., *Barleria lupulina* Lindl., *Coptosapelta tomentosa* (Blume) Vahl.ex Heyne var. dongnaiensis (Pit.) Phamh., *Dischidia rafflesiana* Wall., *Drynaria quercifolia* (L.) J.Sm., *Chromolaena odorata* (L.) syn.: *Eupatorium odoratum* L. King et Robinson, *Ficus pumila* L., *Gleditsia fera* (Lour.) Merr., *Ipomoea biloba* Forsk. syn.: *Ipomoea pes-carprae* (L.) Sweet, *Justicia gendarussa* L. syn.: *Gendarussa vulgaris Nees, Leea rubra Blume, Momordica cochinchinensis* (Lour.) Spreng., *O. indicum* (L.) Vent, *Parabarium micranthum* (Wall.) Pierre ex Spire, *Scoparia dulcis* L. and *Smilax glabra* Roxb. Out of these 17 plants, only the dichloromethane extracts obtained from *Chromolaena odorata* leaves and the stem bark of *O. indicum* showed distinct inhibitory effects on NF-κB activation at a concentration of 10 µg/mL. Based on the findings described in both independent but related studies, we can speculate that *O. indicum* is a highly potential medicinal plant for anti-inflammatory effects via the inhibition of NF-κB signalling pathway.

NF-κB is a transcription factor which regulates multiple aspects of innate and adaptive immune functions and serves as a pivotal mediator of inflammatory responses. It participate in inflammatory response by inducing the expression of various pro-inflammatory cytokines (e.g., IL-1, IL-2, IL-6, TNF-α, etc.), chemokines (e.g., IL-8, MIP-1α, eotaxin, etc.), adhesion molecules (e.g., ICAM, VCAM, E-selectin), inducible enzymes (COX-2 and iNOS), growth factors, and immune receptors [50]. Nowadays, inhibition of NF-κB signalling pathway is established as one of most important targets for the treatment of a wide variety of inflammatory diseases, autoimmune diseases as well as cancers. Therefore, *O. indicum* extract which could significantly inhibit NF-κB should be exploited for future development of anti-inflammatory drugs.

#### 4.1.8. Wound Healing

Similar to anti-inflammatory effects, *O. indicum* plant has been used since ancient days in Ayurvedic medical practice for the treatment of wounds. Wound healing is a complex process of homeostasis, re-epithelialisation, granulation, tissue formation and remodeling of the extracellular matrix that takes place by itself under normal condition. However, there are various risk factors such as infection, aging and bad health conditions can affect or delay the healing process. Wound healing is affected by the cytokines secreted by fibroblasts such as platelet derived growth factor (PDGF), transforming growth factor-β (TGF-β), vascular endothelial growth factor (VEGF), and basic fibroblast growth factor (bFGF) that are required for cell proliferation and formation of new blood vessels in the regenerating wounds [51,52]. These cytokines play a major role in wound healing, in which their deficiency has been reported to retard several important aspects of wound healing including inflammation, fibroplasia, synthesis of proteoglycans and collagen, angiogenesis and wound remodeling [53].

Singh et al. [54] has reported that *O. indicum* root bark extract treated groups showed a better healing pattern with complete wound closure in mice models within 32 days while it was about 40 days in non-treated mice. Moreover, 2.5% methanolic extract treated animals showed faster epithelialization of wound (29 ± 0.3535) than the animals treated with 1% methanolic extract (33 ± 0.2165). This indicated that higher concentration of extract was more effective in wound healing process. The 2.5% methanolic extract also showed signs of advanced healing such as complete restroration of epidermis, well organized and high amount of collagen bundles in dermis and absence of inflammatory cells in fully grown dermis. The hydroxyproline content also was found to increase significantly (29 ± 0.5780) in *O. indicum* treated group as compared to the non-treated group (37 ± 0.6123). High hydroxyproline content indicated increased collagen synthesis to facilitate wound healing. The extract also exhibited antibacterial effects against *B. subtilis, S. aureus, E. coli, Streptococcus* sp. and *Aspergillus niger* that are commonly found in wound infection.

Furthermore, application of *O. indicum* stem bark ethanol extract by Lalrinzuali et al. [4] on deep dermal excision wound also proved the claim that *O. indicum* aids in the wound healing process. Topical application of the extract reduced the mean healing time by approximately eight days for 5% and 10% ethanolic extract when compared to the non-treated group. Wound contraction for the 10% ethanolic extract-treated group was better than the standard drug-treated group and the rise of collagen synthesis was almost 3.5-fold higher for ethanolic extracts treated group when compared to a non-treated group. The DNA and collagen synthesis also increased significantly in the regenerating wound tissue, indicating rise in fibroblast proliferation. This led to earlier closure of the wound and reduced wound healing time. The western blot analyses also revealed that the *O. indicum* extract showed suppression of NF-κB and COX-II (in a dose dependent manner up to 10% ethanolic extract when compared to non-treated control. These reports clearly shown that the increased wound healing ability by *O. indicum* extract could be due to its ability to suppress the expression of genes related to proinflammatory cytokines which would hasten the early healing and closure of excision wounds [4].

### 4.2. Strength and Limitations

This systematic review is among the first to describe in detail the in vitro and in vivo studies on the efficacy of baicalein extracted from *O. indicum* medicinal plant against various human communicable and non-communicable diseases. It prioritises the therapeutic potential of the selected active constituents (particularly baicalein only) whilst various other studies focus on the whole plant crude extract (mixture of different types of active compounds). Thus, this review has a huge advantage in providing the knowledge gap on the baicalein extracted from *O. indicum*, which can be used as a reference for new research to support the efficacy of this particular active compound instead of the mixture of different unidentified active compounds as a novel therapeutic agent against human diseases. This review study also identified several limitations. Several articles reviewed in this study did not clearly state the proper methodology of the plant extraction and the final percentage of baicalein in the extract, and hence restricted future studies to reproduce the same extract. Furthermore, there is still insufficient clinical evidence to draw a definitive conclusion on the efficacy and safety of this active compound for patient usage.

### 4.3. Recommendations

Based on this review, we suggest for more in-depth clinical investigations of this complementary therapy to develop evidence-based medicine in the future. Moreover, detailed consensus on extraction method, phytochemistry as well as the toxicological aspects should be developed to maintain the reproducibility and accuracy of the overall development of this active compound as a novel lead agent for human diseases therapy. Last but not least, we also suggest that conservation of *O. indicum* plant should be done on a large scale through both in-situ and ex-situ methods in order to save this precious plant for the benefit of mankind.

## 5. Conclusions

This systematic review article provides new insight into the traditional medicinal plant *O. indicum* and its active constituent baicalein as a prospective complementary therapy from the perspective of modern and scientific aspect. According to the published data collected in this review, we can reasonably conclude that baicalein extracted from *O. indicum* plant possesses anti-cancer, antibacterial, anti-hyperglycemia, neurogenesis, cardioprotective, anti-adipogenesis, anti-inflammatory, as well as wound healing effects. Some of these effects are direct and some are indirect, and each mechanism of actions must be clearly understood in order to improve the efficacy of this plant in treating diseases. However, there was a scarcity of evidence on the efficacy of this natural product in clinical studies. Therefore, more evidence based clinical studies are required to verify the efficacy and safety of baicalein as potential therapeutic agent for various human diseases.

## Figures and Tables

**Table 1:** The year of publication for the selected articles discussed in this systematic review.

Year	No. of Publication
2010	1
2011	1
2013	3
2015	2
2016	1
2017	3
2018	5
2019	3
2020 (up to 31 March 2020)	1
TOTAL	20

**Table 2:** Summary of key findings of in vitro and in vivo studies using *O. indicum* extract containing baicalein for treatment.

Author, Year	Reference	Biological Activities	Part and Solvent Used	Summary of Key Findings in Vitro Tests	Summary of Key Findings in Vivo Tests
Chassagne, 2018	[16]	Anti-cancer	Stem bark (ethanol extract)	*O. indicum* extract showed significant highest anti-proliferative effect on human liver cancer cell line (HepG2) (IC_50_ = 64.1 μg/mL) compared to extracts isolated from the other nine types of Southeast Asian plants such as *Andrographis paniculata* (Burm.f.) Nees, *Willughbeia edulis* Roxb, *Senna alata* (L.) Roxb., *Cananga latifolia* (Hook.f. & Thomson) Finet & Gagnep., *Salacia chinensis* L., *Orthosiphon aristatus* (Blume) Miq., *Boerhavia diffusa* L., *Gomphrena celosioides* Mart. and *Melastoma saigonense* (Kuntze) Merr.	Not available (N/A)
Yang, 2018	[17]	Anti-cancer	Not mentioned (the baicalein from *O. indicum* was given as a gift by other research group)	Baicalein isolated from *O. indicum* plant significantly induced apoptosis of bladder cancer cells through the inhibition of anti-apoptotic genes such as BCL-2, Bcl-xL, XIAP and surviving after 72 h treatment at doses of 5 mg/mL.	N/A
Kang, 2019	[11]	Anti-cancer	Leaves (petroleum ether-methanol extract)	Baicalein-enriched fraction extracted from the leaves of *O. indicum* possessed higher baicalein content, stronger anti-oxidant activity and enhanced efficiency to treat human GBM brain cancer, as compared to crude extract which contained lesser baicalein content (IC_50_ baicalein-enriched fraction = 36 μg/mL vs. IC_50_ crude extract = 92 μg/mL).	N/A
Wahab, 2018	[26]	Anti-cancer	Leaves (methanol extract)	Baicalein-enriched fraction extracted from the leaves of showed higher potential to inhibit cervical cancer (SiHa and HeLa cell lines), as compared to the *O. indicum* crude extract and cisplatin. Western blot analysis showed that baicalein-enriched fraction-treated SiHa and HeLa cells exhibited anti-HPV effects through the down-regulation of E6 and E7 oncoproteins that play a key role in human papillomavirus (HPV)-associated cervical carcinoma tumorigenesis.	N/A
Zazali et al., 2013	[28]	Anti-cancer	Leaves (methanol extract)	Methanolic leaf extract of *O. indicum* at concentration of 3.87 μg/mL inhibited cervical cancer (HeLa cell line) growth by inducing G1/S cell cycle arrest and apoptosis via p53-mediated pathway.	N/A
Lalou, 2013	[30]	Anti-cancer	Stem bark (methanol extract)	Baicalein inhibited kexin-type proteases of superfamily proprotein convertase subtilisin/kexins 3 (PCSK3) more efficiently (IC_50_ = 254.1 μM) compared to other flavonoid found in *O. indicum* such as chrysin, oroxylin-A, oroxylin-A glycoside and baicalin. PCSK3 also known as furin. It plays role to cleave and convert inactive growth factors into their matured forms that promote tumor growth. Thus, PCSK3/furin inhibitor could inhibit cancer progression to a significant extent. These findings justify and rationalize the potential of baicalein as a new PCSK3/furin inhibitor for future cancer therapy.	N/A
Sithisarn, 2016	[7]	Antibacterial, antioxidant	Fruits (ethanol and water extract)	*O. indicum* ethanol extract at concentration 1000 mg/mL exhibited intermediate antibacterial activity against *S. intermedius* and *S. suis* with an inhibition zone of 15.11 mm and 14.39 mm, respectively. It also showed stronger in antioxidant activities with EC_50_ value of 26.33 µg/mL, compared to water extract.	N/A
Sithisarn, 2019	[12]	Antibacterial	Fruits (ethanol and water extract)	*O. indicum* fruit ethanol extract promoted the stronger antimicrobial activity against four clinical pathogenic bacteria, including *S. intermedius* (IC_50_ = 1.30 mg/mL), *S. suis* (IC_50_ = 7.81 mg/mL), *P. aeruginosa* (IC_50_ = 39.20 mg/mL), and *β-E. coli* (IC_50_ = 66.85 mg/mL), compared to water extract on *S. intermedius* (IC_50_ = 7.81 mg/mL), *S. suis* (IC_50_ > 250 mg/mL), *P. aeruginosa* (IC_50_ > 125 mg/mL), and *β-E. coli* (IC_50_ > 250 mg/mL). Ethanol extract showed higher potential as an antibacterial agent than the water extract because the flavonoid content (including baicalein) was found significantly higher in the ethanol extract compared to water extract (based on the optimized and validated HPLC data).	N/A
Singh & Kakkar, 2013	[34]	Anti-hyperglycemia, antioxidant	Stem bark (50% aqueous ethanol)	In vitro testing of *O. indicum* extract was done using mature 3T3-L1 adipocyte cell line. Data indicated that *O. indicum* extract treatment exhibited strong antioxidant capacity and potential to inhibit α-glucosidase activity and bovine serum albumin (BSA) glycation comparable to clinically used anti-diabetes drugs such as acarbose. Besides, the extract treatment also was found to improve insulin sensitivity in the 3T3-L1 adipocyte cells.	In vivo testing of *O. indicum* extract was done using streptozotocin induced type II diabetic rats. Feeding of *O. indicum* extract of 250 mg/kg bwt via oral administration was found to improve the rats antioxidant status (*p* < 0.01).
Zhang, 2017	[39]	Anti-hyperglycemia	Seeds (90% aqueous ethanol)	A combination of *O. indicum* seed extract and acarbose drug synergistically reduced postprandial blood glucose (PBG) through inhibition of rat intestinal α-glucosidase activity.	At doses between 50 and 200 mg/kg, *O. indicum* extract enhanced the efficacy of acarbose in controlling blood glucose level in diabetic mice by up to 5-fold.
Sun, 2017	[36]	Anti-hyperglycemia	Seeds (90% ethanol-water)	N/A	The combined drugs of acarbose and an *O. indicum* seed extract can reduce the dose of acarbose by 80% and reduce the risk of diabetes by 75% in prediabetic mice. The glucose levels of acarbose + *O. indicum* group were significantly lower than *O. indicum* only group and acarbose only group. These data demonstrated that the acarbose + *O. indicum* exhibited a reduced risk of prediabetes progressing to diabetes compared to sole acarbose or *O. indicum* treatments. Also, the combined *O. indicum* + acarbose treatment showed lower toxicity effects on the prediabetic mice, indicated by lower alanine aminotransferase (ALT) level, lesser liver cellular swelling and hepatic steatosis as compared to acarbose single treatment group.
Fuentes, 2015	[44]	Neurogenesis	Bark (methanol)	The crude methanol extract of *O. indicum* (100 lg/mL) induced a two-fold increase and activated neurogenin 2 (Ngn2) promoter activity. Ngn2 is a type of basic helix-loop-helix (bHLH) activators which play roles in neurogenesis by inducing the differentiation of neural stem cells (NSCs) into matured neurons.	N/A
Menon, 2019	[45]	Cardioprotective	Root bark (70% methanol)	N/A	*O. indicum* extract is capable to prevent damaging and devastating effects of cumulative administration of doxorubicin (30 mg doxorubicin/kg of animal body weight) in Sprague-dawley (SD) rat models.
Tanaporn, 2018	[46]	Anti-adipogenesis	Fruit pods (ethanol extract)	Treatment with 200 μg/mL *O. indicum* fruit pod ethanolic extract significantly decreased the intracellular lipid accumulation by approximately 52%, compared to the non-treated adipocyte (control). The extract also showed potential to inhibit pancreatic lipase activity. More importantly, the researchers also confirmed the non-toxicity effect of this extract on pre- and mature adipocytes at low doses (50 to 200 ug/mL), assuring the safety of taking this traditional supplement as a diet plan for the management of overweight or obese.	N/A
Tanaporn, 2020	[55]	Anti-adipogenesis	Fruit pods (ethanol extract)	*O. indicum* extract at 200 μgmL^−1^ exhibited anti-adipogenesis effects via the downregulation of adiponectin, a hormone that plays crucial role in adipogenic process. Evaluation of mRNAs expression profile revealed that *O. indicum* extract treatment significantly inhibited the expression of PPARγ2 and SREBP-1c and lowered the level of expression of GLUT4, FAS, and leptin compared to the non-treated control (*p* < 0.05).	N/A
Mangal et al., 2017	[48]	Anti-adipogenesis	Bark (ethyl acetate extract)	Ethyl acetate extract of *O. indicum* bark was found to be the most potent extract for inhibition of pancreatic lipase enzyme and adipogenesis in fat tissue compared to other five plant species namely: *Limonia acidissima* Groff (Kapittha)*, Cassia siamea* Lam. (Kassod)*, Swertia chirayita* (Roxb.) Buch.-Ham. (Kiratatikta), *Carissa carandas* Linn. (Karamarda) and *Capparis decidua* (Forssk.) Edgew. (Karir).	N/A
Siriwatanametanon et al., 2010	[49]	Anti-inflammatory	Stem bark (methanol extract)	Nine traditional plants with uses linked to anti-inflammatory as recorded in Thailand formal textbook were evaluated in this study. Among all the nine species, only *Gynura pseudochina var. hispida* and *O. indicum* showed the most promising NF-κB inhibitory effects with the lowest IC_50_ values (41.96 and 47.45 μg/mL, respectively). NF-κB is a transcription factor which regulates multiple aspects of innate and adaptive immune functions and serves as a pivotal mediator of inflammatory responses. Inhibition of NF-κB can significantly inhibit inflammatory response.	N/A
Tran et al., 2015	[15]	Anti-inflammatory	Stem bark (petroleum ether, ethyl acetate and methanol extract)	17 medicinal plants used in Vietnamese traditional medicine for the treatment of inflammatory disorders were evaluated in this study. Out of these 17 plants, only the dichloromethane extracts obtained from *Chromolaena odorata* leaves and the stem bark of *O. indicum* showed distinct inhibitory effects on NF-κB activation at a concentration of 10 µg/mL.	N/A
Singh, 2011	[54]	Wound healing, antimicrobial	Root bark (methanol extract)	N/A	*O. indicum* extract had significant wound healing activity as evident from the rate of wound contraction. Highly significant wound contraction was observed from day 20 onward in *O. indicum* treated animal groups. The period of epithelization also decreased significantly and higher hydroxyproline content in treated groups suggested higher collagen re-deposition than the control group. The extract also exhibited antibacterial effects against *B. subtilis*, *S. aureus*, *E. coli*, *Streptococcus* sp. and *Aspergillus niger* that are commonly found in wound infection.
Lalrinzuali, 2018	[4]	Wound healing	Stem bark (ethanol)	N/A	Topical application of different concentrations of *O. indicum* extract resulted in a concentration dependent rise in wound contraction and reduce in mean wound healing time. It also increased the DNA and collagen syntheses in a dose dependent manner at all post wounding days and the greatest acceleration in DNA and collagen formation was observed for 10% *O. indicum* extract. The study of molecular mechanisms revealed the suppression of NF-κB and COX-II in a dose dependent manner in the regenerating wound of mice with a maximum inhibition using 10% *O. indicum* extract.
